# Influence of Surface Treatments and Adhesive Type on Bond Strength Between Stainless Steel and CFRP in Agricultural Machinery

**DOI:** 10.3390/ma18133027

**Published:** 2025-06-26

**Authors:** Leif Steuernagel, Carsten Schmidt, Christian Jenensch

**Affiliations:** 1 Institute of Polymer Materials and Plastics Engineering, Clausthal University of Technology, Agricolastraße 6, 38678 Clausthal, Germany; leif.steuernagel@tu-clausthal.de; 2 Institute of Production Engineering and Machine Tools, Leibniz Universität Hannover, Ottenbecker Damm 12, 21684 Stade, Germany; schmidtc@ifw.uni-hannover.de; 3 Institute of Polymer Materials and Plastics Engineering, Clausthal University of Technology, Ottenbecker Damm 12, 21684 Stade, Germany

**Keywords:** metal–CFRP joint, adhesive bonding, surface treatment, surface roughness, lightweight agricultural structure, carbon fiber chassis, adhesive interface analysis

## Abstract

In the domain of agricultural machinery, the utilization of carbon fiber-reinforced plastics (CFRP) for structural components, such as the chassis, facilitates substantial weight reduction. To integrate additional components, stainless-steel connection points can be bonded to the CFRP chassis using adhesives. This study investigates surface preparation methods to enhance adhesive bonding strength at the coupon level. Three adhesives (DP490, MA8110, SG300) were tested on untreated, sandblasted, and sandpaper-grinded steel surfaces. Contrary to predictions, the highest strength (28.7 MPa) for DP490 was achieved after simple acetone cleaning, despite lower surface roughness (Ra = 1.60 µm), while sandblasting (Ra = 3.71 µm, 22 MPa) and grinding (Ra = 2.78 µm, 25.95 MPa) performed worse due to incomplete adhesive penetration. Subsequent tests on DP490 with laser structuring (Ra = 88.8 µm) and sandblasting with coating (Ra = 1.94 µm) provided strengths of 27.5 MPa and 29.3 MPa, respectively. The findings indicate that, under the examined conditions, surface cleanliness plays a more critical role in adhesive bonding strength than surface roughness. Practically, acetone cleaning is a cost-effective and time-efficient alternative to treatments like sandblasting or laser structuring. This makes it attractive for industrial use in agricultural machinery. While this study focuses on coupon-level surfaces, the findings provide a basis for scaling to component-level applications in future research.

## 1. Introduction

The development of lightweight structures for heavy-duty vehicles, such as agricultural machinery, has become a major focus due to growing concerns about global energy and climate issues, as well as regulatory approval, weight limits, and soil compaction. In this context, the chassis represents a central component of every agricultural machine and is traditionally constructed of steel, which offers certain advantages. The introduction of alternative lightweight materials, such as carbon fiber-reinforced plastics (CFRP), has already engendered new opportunities for innovation in a variety of other sectors, including aerospace and automotive industries [1,2,3]. Consequently, the utilization of such materials in agriculture machinery is a promising approach to lightweight construction.

**Figure 1:**
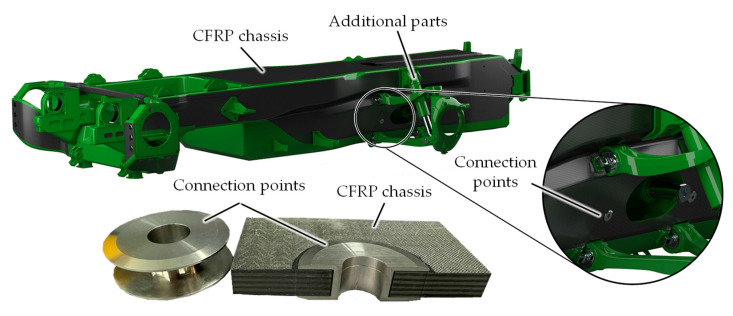
CFRP chassis with additional parts and connection points.

The chassis, being the central component within the structure, must be capable of absorbing all static and dynamic loads. Typically, additional parts, such as water tanks or wheel suspensions, are attached to the chassis using bolt connections, welding, or screwing. However, this presents a problem in terms of integrating a lightweight chassis, since the methods employed for the attachment of these components are not readily transferable to a CFRP chassis. This poses a significant challenge in terms of force application, particularly in the context of handling heavy loads. In order to address this issue, steel connection points were developed (Figure 1). The purpose of these points is to act as an interface between the conventional steel attachments and the fiber composite structure, facilitating bolt connections.

In most of the commercially available joining techniques, such as bolting, a metal element is coupled with a common joining element and placed into the CFRP. This attachment element creates a detachable joint, simplifies the joining of several parts, and allows for the inspection of the joint [4]. For CFRP in particular, fasteners destroy the integrity of the carbon fiber, and the presence of holes causes stress concentration. Given that the design of the chassis incorporates holes for bolt connections, it is imperative that the connection points are attached to the chassis without introducing additional weakening.

Adhesive bonding has been shown to have a number of advantages over mechanical joining. These include the fact that the weight of the fasteners used is minimal compared to bolts or screws. Furthermore, adhesive bonding is capable of high energy absorption and offers design flexibility. One of the most significant advantages of adhesive bonding is that it allows the combination of materials with varying physical and geometric characteristics without causing any alterations to the structure of these materials [4,5,6]. Therefore, adhesive bonding has become one of the most common joining techniques for steel and CFRP in recent years [5,7,8].

**Figure 2:**
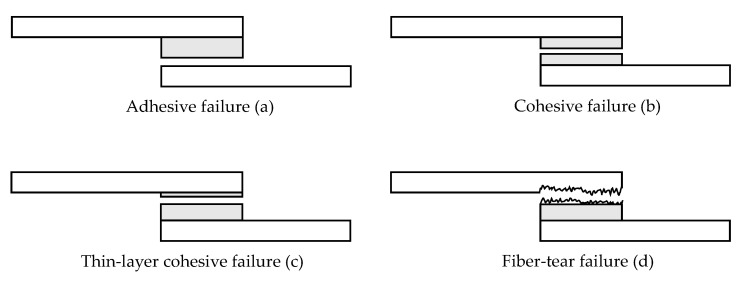
Representation of typical failure modes in steel composite bonded joints.

The selection of an adhesive for this particular joint is a complex process. The bonded joint must fulfil several criteria, including high adhesive strength between steel and CFRP, a specific bonding gap resulting from the connection point geometry, and the necessity of curing at room temperature. In metal–CFRP bonded joints, failure is more likely to occur at the metal/adhesive interface than at the CFRP/adhesive interface due to the greater differences in physical and chemical properties between the metal and adhesive [5,7,9]. For this reason, the analysis of suitable surface treatments for steel and the analysis of the failure mode are of particular importance and essence. According to ASTM D5573-99(2019) [10], six types of failure modes exist for composite joints, but in metal–plastic bonding, this is typically reduced to the following four: (a) adhesive failure, (b) cohesive failure, (c) thin-layer cohesive failure (mixed mode), and (d) fiber-tear failure (substrate failure) (Figure 2) [11,12,13]. In the case of adhesive failure, a fracture occurs at the interface between the adhesive and the bonded part. Cohesive failure, on the other hand, is a breakdown of the intermolecular bonding forces in the adhesive substance. Mixed failure occurs when the crack propagates both at the interface and in the adhesive. Substrate failure indicates that both the interface and adhesive are stronger than the adherend. Although substrate failure is ideal, cohesive failure is generally preferred [5]. However, for all practical purposes, the adhesive is more likely to fail during application because of the poor interfacial adhesion between the adhesive and the substrate. In particular, when bonding steel to CFRP, the steel/adhesive interface has been found to be fragile and therefore prone to adhesive failure [14]. Consequently, enhancing the interfacial adhesion between substrates and adhesives is an important issue.

One method of enhancing the interfacial bond strength is the implementation of surface treatments, which are applied to the metal or CFRP surface. In recent decades, many surface treatment methods have been developed, including mechanical treatment such as burnishing [15], sand or grit blasting [9,16,17], hand grinding (e.g., with sandpaper) [18,19], chemical treatment [20,21,22], and laser treatment [5,23,24]. Mechanical treatment with sandpaper is a simple and practical method to achieve the desired surface characteristics. The manner in which sandpaper is used to sand a surface can result in the formation of different surface structures. Yang et al. [18] investigated the effect of the sandpaper direction on the bonding strength when sanding CFRP. It was shown that the bonding strength can be improved by sanding the surface. It was also found that the highest bond strength can be achieved with random sanding directions. Teng et al. [25] investigated the effect of surface preparation on the performance of CFRP–steel bonds using sandblasting and demonstrated that surface preparation can enhance bond strength. Furthermore, Tavakkolizadeh et al. [26] proposed that sandblasting is an effective method for surface preparation. Additionally, laser treatment of CFRP is a prevalent technique to enhance bond strength. CO_2_ laser texturing at different densities on CFRP was performed by Sorrentino et al. [27] to improve the bond strength, which was explained by the increase in surface roughness. Zou et al. [5] also investigated different overlap rates of laser spot and pulse fluence on steel–CFRP bonds. Increasing the pulse fluence on the steel surface resulted in an increase in roughness, which reduced the contact angle and improved the surface energy. This phenomenon leads to a better mechanical interlocking effect, as well as improving the surface wettability of the adhesive to the steel surface.

Moreover, studies have demonstrated that plasma or chemical treatment can influence the surface chemical composition and surface morphology [28,29]. Consequently, this can enhance the bonding strength between the adhesive and the steel. However, it was not possible to entirely avoid interfacial failure between steels and adhesives. In addition, there are a number of issues associated with mechanical, plasma, and chemical treatment methods. These include certain processes, such as laser treatments, that are complex, expensive, and ecologically detrimental. Consequently, their utilization within industrial contexts is constrained [5].

An additional element that must be taken into consideration when assessing the adhesive bond is its environmental resistance, especially when used in harsh or changing outdoor conditions, and the operational stresses, as in the case with agricultural machinery. These machines are often exposed to direct sunlight and have components that generate heat, such as the engine. These elevated temperatures can significantly affect the mechanical integrity of CFRP-to-metal bonds, particularly due to temperature-induced degradation of the adhesive system. In comparison with applications at lower temperatures, adhesive bonding between steel and CFRP becomes significantly more critical at elevated temperatures. This increased sensitivity is primarily due to the pronounced reduction in the strength and stiffness of the resin or adhesive as the temperature surpasses its glass transition temperature (Tg), which typically lies below 100 °C for most cold-curing resin and adhesive systems [30,31]. In the study conducted by Al-Shawaf et al. [32], three epoxy adhesives that were commercially available were utilized to fabricate steel/CFRP double-strap joints. The mechanical responses of these joints in tension were examined at temperatures of 20, 40, and 60 °C. A decline in bond strength was observed, ranging from 20 to 30%. CFRP rupture at the bond region was identified as the predominant failure mode for the 20 and 40 °C exposures. Conversely, debonding failure emerged as a pervasive trend for joints subjected to 60 °C conditions. It has been demonstrated that, within the context of steel/CFRP double-strap joints exposed to elevated temperatures, the mechanical performance is predominantly influenced by the adhesive component.

In addition to elevated temperatures, ultraviolet (UV) radiation must also be considered, given its significant role in the outdoor exposure of agricultural machinery. Nguyen et al. [33] investigated the influence of UV exposure on CFRP–steel double-strap joints, observing a strength reduction of up to 18.7% after 372 h of UV irradiation per surface. However, further analysis indicated that the adhesive layer may not have been directly affected by UV radiation due to its encapsulation between the CFRP and steel. In contrast, the prevailing thermal effects, approximately 40 °C during UV exposure, were determined to constitute the primary factor responsible for the degradation process. This finding indicates that temperature remains the predominant factor in the degradation of adhesive bonds under combined environmental loads, particularly when the adhesive is shielded from direct UV exposure.

Alongside thermal and UV influences, moisture represents another critical environmental factor for CFRP-based structures. Humidity often has a combined effect with temperature, and an elevated temperature appears to accelerate the degradation of material induced by moisture [34]. Consequently, a hot, wet environment is regarded as more aggressive, yet still realistic, for agricultural machinery. De Nève et al. [35] examined the effects of humidity on an epoxy adhesive and small-scale aluminum–steel adhesively bonded joints at a constant temperature of 70 °C. They found that the Tg of the adhesive decreased with the moisture uptake and that the measured adhesive shear modulus showed a reduction in correlation with exposure time. These results are likely to be related to the plasticization of the polymer, as also discussed by Bowditch et al. [36] and Knox et al. [37].

In this paper, three adhesives were initially analyzed and compared based on the specific requirements for joining steel connection points to a CFRP chassis. Furthermore, the potential of various surface treatments to enhance the adhesive performance of these steel elements was evaluated. Five methods were investigated, resulting in the testing of 66 specimens. The objective was to evaluate the impact of different surface treatments, in conjunction with three industrially relevant adhesives, on the bond strength between stainless steel and CFRP. The analysis will identify processable and robust adhesives suitable for future component-level applications in agricultural machinery.

## 2. Materials and Methods

### 2.1. Materials

The unidirectional carbon fiber textile utilized in this study was the HPT 620 C0 SF6090 from Kümpers GmbH (Rheine, Germany), with a grammage of 620 g/m^2^. The epoxy resin system Polyvertec 3451 with amine hardener H1 from Schill + Seilacher GmbH (Hamburg, Germany) was selected as the matrix for the CFRP. The CFRP sheets are composed of three layers of carbon fiber, with the plies oriented at 0°/90°/0°. This results in a thickness of approximately 1.8 mm. A two-millimeter-thick plate of alloy 1.4301 was selected for the stainless steel. For both materials, a geometry of 25 mm by 100 mm was utilized. To bond the two materials, three distinct adhesives were used, as illustrated in Table 1.

Both the DP490 and the MA8110 adhesives are known for their high strength and good toughness. The optimal strength of the DP490 adhesive is achieved when the gap is within the range of 0.05 and 0.15 mm. In contrast, the SG300 adhesive demonstrates comparatively low strength but high toughness at a minimum gap of 0.5 mm, while the MA8110 is suitable for bonding gaps ranging from 0.75 to 12.7 mm. For both adhesives, MA8110 and SG300, light surface preparation is advertised, depending on the material pairing. However, for DP490, sanding with at least 180- to 360-grit sandpaper is strongly recommended.

### 2.2. Methods

#### 2.2.1. Surface Preparation and Measurement

Five different surface treatments were applied before bonding in order to investigate the possibility of increasing the interfacial adhesion between the steel surface and the adhesives. The steel plates were pretreated using sandpaper grinding (SG) with 80-grit sandpaper, sandblasting (SB) with glass beads with a diameter of 1 mm, the sandblast coating method (SACO—SA), and simple cleaning with acetone (SC). The possibility of structuring the surface with a laser was also investigated as a further surface treatment (LS).

The surface roughness was measured using confocal laser microscopy, whereby a VK-X1000 laser scanning microscope from Keyence Deutschland GmbH (Neu-Isenburg, Germany) with 5× magnification was utilized. Due to the fact that impurities on the surface can be problematic when determining roughness, the stainless-steel surface was cleaned with acetone and then cleaned of all impurities using compressed air. The arithmetic mean R_a_ of the absolute vertical deviation values of the roughness profile from the mean line was then analyzed using DIN EN ISO 4288:1998-04 [38] and ASME B46.1:2019 [39,40]. In each batch, three specimens were examined at three different positions. Thus, overall, nine measurement results were obtained for each batch, which were then used to calculate the corresponding mean values and standard deviations for data evaluation.

#### 2.2.2. Specimen Manufacturing, Processability, and Tensile Lap-Shear Testing

The investigation concentrated on the surface treatment of the steel, as it was anticipated that failure would occur at the interface of the steel surface and the adhesive. Consequently, the CFRP panels were meticulously cleansed with isopropanol. Following the application of the surface treatments, all samples, including the received SA and LS samples, were first cleaned by immersion in acetone for a period of five minutes. Afterwards, the samples were dried with the use of compressed air to remove any residual particles. To prevent sample shifting, a bonding tool was designed and manufactured. Positioning pins and alignment guides were used to create a consistent bonding surface and ensure the reproducibility of the specimen geometry.

**Figure 3:**
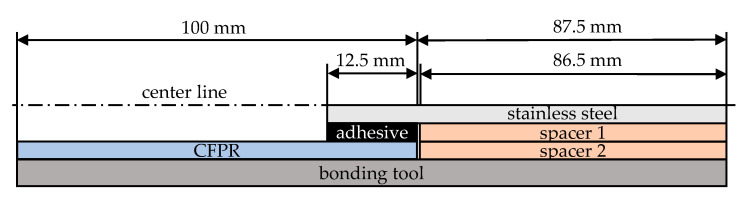
Schematic illustration of the bonding process with spacers.

In the first step, the CFRP part was placed on one of the guides. The metal part was then bonded to the first CFRP part with a bonding gap of 0.5 mm, a consequence of the geometry of the connection point. The adhesive was applied in accordance with the manufacturer’s instructions. For the two-component epoxy adhesives, a dispensing gun equipped with a static mixing nozzle was used to ensure homogeneous mixing and precise application. The adhesive was applied across the entire bonding area of both sample parts, thereby ensuring complete surface coverage. Following the placement of the components, excess adhesive was carefully removed using a wooden stick to achieve clean edges and a smooth bonding interface. To ensure consistent bond thickness and proper alignment during the curing process, a 2 kg weight was positioned on top of each sample, with spacers used to maintain a consistent distance between the parts. Each spacer had the same thickness as the respective part and the bonding gap (Figure 3). The load was applied to prevent misalignment between the CFRP and steel surfaces, with the weights remaining in position throughout the entire curing process to improve reproducibility and bond quality. All samples were subjected to a curing process in a climate-controlled room, the temperature of which was maintained at 20 ± 2 °C and the relative humidity at 45 ± 5%. This ensured consistent environmental conditions for all adhesive systems throughout their respective curing periods. Subsequent to the curing of the initial bond, the second CFRP component was bonded to the metal component employing the identical procedure and environmental conditions.

To verify the actual bond gap thickness, dimensional measurements were performed before and after bonding. Prior to bonding, all individual sample components were measured at both ends and in the center for thickness. After each bonding step, the resulting thickness of the bonded assembly was measured at the bonding locations. The bonding gap was then calculated by subtracting the sum of the individual component thicknesses from the total assembly thickness, providing quantitative verification of the achieved bond gap thickness.

In order to evaluate the processability of the three adhesives, a comparative analysis was conducted in two areas: firstly, with regard to the adhesive preparation, and secondly, the actual bonding process. The analysis of the adhesive preparation focused on the individual process steps required for the application of the adhesive, with particular attention paid to the storage and mixing of the adhesive. The bonding process was then analyzed in terms of the handling of the adhesive, with particular attention paid to the behavior of the adhesive on the substrate surfaces and the processing time.

**Figure 4:**
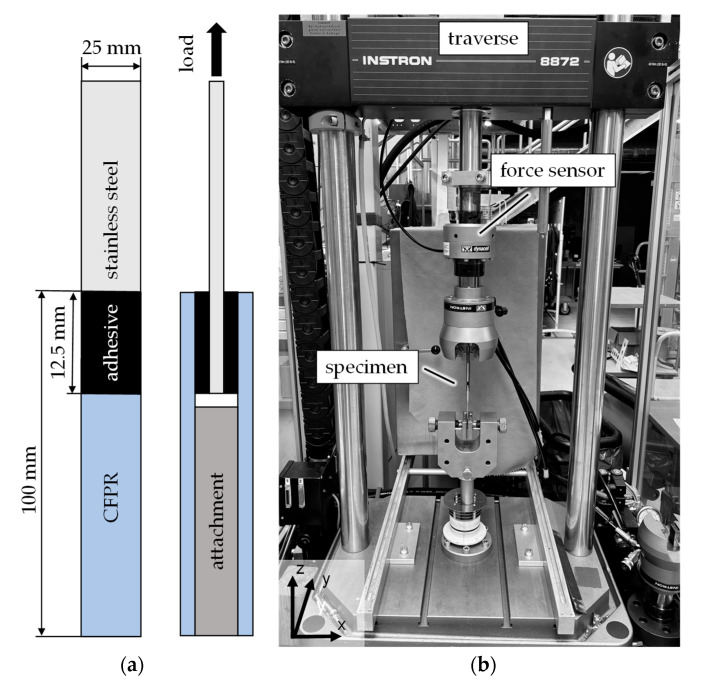
(**a**) Schematic diagram of adhesive-bonded specimen, and (**b**) experimental setup of the tensile lap-shear test.

In this study, double-lap-shear specimens were selected in order to minimize bending moments and stress concentrations at the adhesive interface [41,42]. Compared to single-lap configurations, the symmetrical design of double-lap specimens results in a more uniform stress distribution across the bond line, significantly reducing the effects of peeling and bending during tensile loading. The testing samples contain two bonding surfaces, each measuring 12.5 mm in length and 25 mm in width (Figure 4a). It is important to note that, in contrast to the sample type and the test setup, the sample geometry and the experimental setup of the tensile lap-shear test are oriented at the standard DIN/TS 54404:2020-10 [43]. In this regard, the chosen double-lap specimen’s configuration is different from the standard recommended single-lap specimen. To create a reliable data basis, six specimens were prepared per combination of surface treatment and adhesive, allowing for possible specimen failures while maintaining at least five valid results per group.

The specimens were clamped at each end between grips of an Instron 8872 Servohydraulic Dynamic Testing System (Norwood, MA, USA) (Figure 4b). To prevent bending due to clamping forces, an attachment was used between the two outer CFRP parts. This attachment was the same material and thickness as the steel component, with additional components representing the adhesive thickness.

The testing process was conducted at a speed of 2 mm/min under standard conditions, with a temperature of approximately 21 °C and a relative humidity of approximately 50%. The lap-shear strength was calculated using Equation (1).

(1) $$\tau =\frac{F_{max}}{2\cdot A}$$

Given the relatively high cost of the SA and LS surface treatments, the adhesives were initially only compared with the SC, SG, and SB treatments. The adhesive that was deemed most suitable for the intended application was then subjected to testing with the SA and LS treatments.

## 3. Results and Discussion

### 3.1. Processability and Surface Roughness

The process analysis of the three two-component adhesives revealed no significant differences in terms of their adhesive preparation. The adhesives are supplied in cartridges and, according to the datasheets, can be stored at temperatures ranging from 13 °C to 25 °C (DP490/MA8110) or 27 °C (SG300). The bonding process exhibited minimal variation, with the application of the adhesive substrate being consistent across all three adhesives. However, notable differences were observed in terms of viscosity, with MA8110 and SG300 exhibiting lower viscosity compared to DP490. The higher viscosity of DP490 facilitated the positioning of the components, thereby contributing to a slight simplification of the bonding process. In conclusion, it can be stated that the adhesives exhibit minimal differences in terms of their processability, rendering all of them suitable for the industrial bonding of the connection points to the CFRP chassis.

**Figure 5:**
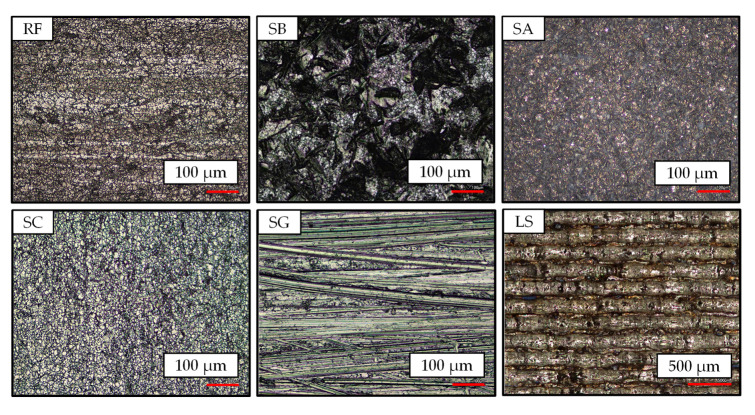
Surface morphology of the steel after different treatments.

The surface morphologies of the steel produced by the five surface treatment methods are shown in Figure 5, where the untreated reference surface is designated as RF. 

It is evident that both the RF and SC surfaces are predominantly smooth, exhibiting a few voids and dents resulting from the manufacturing process. While both surfaces differ solely in terms of the extent to which impurities have been removed, other surface treatments exhibit distinct characteristics. For instance, the SG surface reveals varying depths of oriented grooves caused by the sandpaper. The sanding direction, in the y-direction, is clearly discernible. In the case of sandblasting, irregular roughness features were observed on the steel surface, which were attributed to plastic deformation. In particular, the surface of SB was found to be rougher than that of the SG, with the valleys exhibiting a depth that was more than twice as large. In contrast, the SA surface exhibits a smooth texture, attributable to the additional coating. Finally, the LS surface displays distinctive indentations, caused by the laser’s high power and pulse fluence. This process enables the melting of the steel surface and the subsequent application of a recurring structure. The resultant grooves, with an approximate depth of 0.3 mm and width of 0.1 mm, are distinctly recognizable.

**Figure 6:**
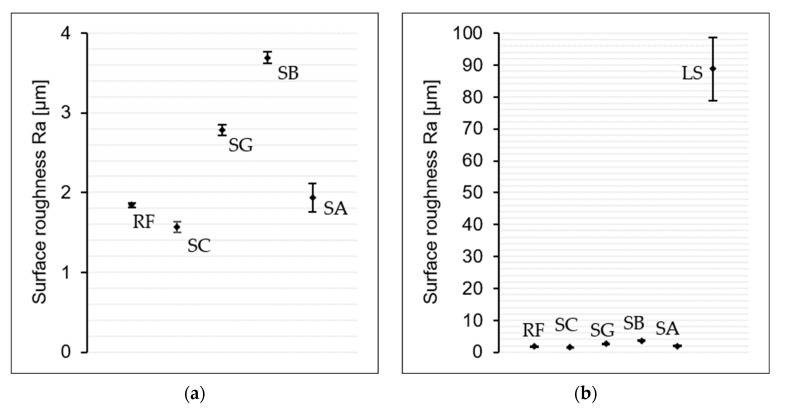
Surface roughness analysis of steel specimens with various surface treatments. (**a**) Detailed view (0–4 µm range) and (**b**) complete data range illustrating the contrast among the four treatments with the LS treatment.

As demonstrated in Figure 6, the surface roughness of all surface treatments is displayed, with the additional RF surface. It can be seen that the surface roughness of the SC is reduced from 1.84 ± 0.04 µm to 1.60 ± 0.07 µm as a consequence of the cleaning process in comparison with the RF surface. This outcome can be attributed to the elimination of dust and other minor particulates on the surface. Conversely, the surface roughness of the SG surface increases from 1.84 ± 0.04 µm to 2.78 ± 0.14 µm, while the SB surface exhibits a twofold increase to 3.71 ± 0.15 µm. In contrast, the SA treatment, resulted in a mere 5% increase in surface roughness, measuring 1.94 ± 0.34 µm. However, the greatest increase in roughness was observed on the laser-structured surface. The LS treatment increased the roughness to 88.8 ± 12.2 µm. The results were to be expected, as sanding with sandpaper, for example, produces numerous scratches; however, compared to sandblasting, the scratches were not deep or rough enough to further increase the surface roughness. The fact that the surface after the SA process is only slightly rougher than the untreated surface was also to be expected due to the additional coating of the surface.

### 3.2. Lap-Shear Strength and Failure Modes

**Figure 7:**
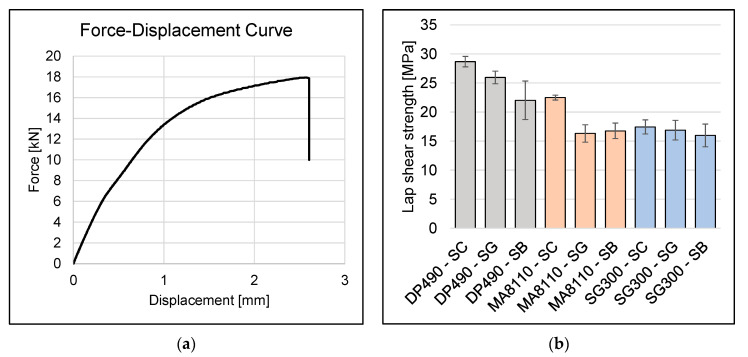
(**a**) Representative force–displacement curve for DP490 adhesive with surface cleaning treatment, specimen 5, and (**b**) tensile lap-shear strength of adhesive-bonded joints with different surface treatments.

The mechanical behavior of the adhesive bonds was evaluated through tensile lap-shear testing. Figure 7a illustrates one example of the DP490 adhesive force–displacement curves obtained from the specimens tested under quasi-static loading conditions. It can be observed that the curve shows an initial linear elastic region, followed by a non-linear response as the force increases to approximately 18 kN at a displacement of about 2.6 mm. The abrupt vertical drop in force at this point indicates a failure of both adhesive bonds simultaneously, which is particularly noteworthy as it suggests that both bonded interfaces experienced nearly identical stress distributions and failed at the same moment. The symmetrical failure mode observed here is advantageous from an analytical perspective, as it indicates that both bonds were subjected to similar loading conditions, thus providing reliable data for determining the shear strength of the adhesive connection.

The tensile lap-shear strength was then determined for all tested specimens with SA, SG, and SB treatments, and the results are summarized in Figure 7b. The results demonstrate that the DP490 adhesive exhibits the highest level of strength. This finding was consistent with the data sheet predictions. Additionally, it can be seen that both DP490 and MA1180 adhesives achieve the highest tensile shear strengths exclusively when surfaces are clean. In contrast, SG300 exhibits comparable performance across all three surface treatments. However, it is observed that MA8110 exhibits a tensile lap-shear strength of 22.5 ± 0.43 MPa, which is 2.8 MPa higher than the expected 19.3 MPa. In contrast, DP490 exhibits a tensile shear strength of 28.7 ± 0.9 MPa, which is 7.1 MPa below the stated value of 36 MPa. However, this discrepancy can be attributed to a nearly fivefold increase in the bonding gap compared to the optimum gap.

The observed outcome was not anticipated in relation to the aforementioned studies on SG [21] and SB [25]. A plausible hypothesis to explain this phenomenon is that the enhanced roughness resulted in the formation of deeper surface depressions, which were not completely penetrated by the adhesive due to its comparatively high viscosity. This may have led to micro-voids or incomplete wetting, ultimately resulting in premature adhesive failure at the metal interface. While elevated curing temperatures could reduce adhesive viscosity and improve wetting, this approach would conflict with the study’s focus on room-temperature processing.

**Figure 8:**
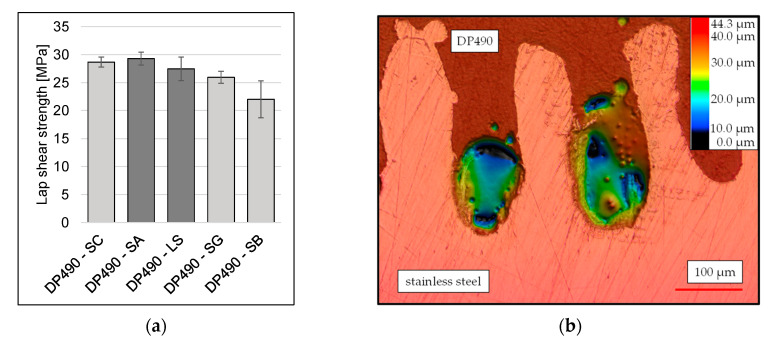
(**a**) Tensile lap-shear strength of DP490 with different surface treatments and (**b**) void between DP490 adhesive and steel after the LS surface treatment.

Following a comprehensive evaluation of the initial tensile shear strength, DP490 was selected for the SA and LS treatment tests. As demonstrated in Figure 8a, the attainable tensile lap-shear strengths reveal that the mean tensile shear strength values obtained for the LS treatments neither increase nor significantly reduce the strength in comparison to SC, while the SA treatment exhibits a slight increase over SC to 29.3 ± 1.16 MPa. However, given the overlapping standard deviations and the close proximity of the mean values, it can be assumed that the differences among the three surface treatments are not statistically significant.

The comparable values of SA and SC can be attributed to the similarity in surface texture. Microscope images reveal that, in the case of the LS treatment, the adhesive was not able to fully penetrate all of the grooves present on the laser-structured steel surface (Figure 8b). As illustrated in the figure, it is evident that specific valleys remain only partially filled or are even unbonded, as indicated by the dark blue to black regions, which in this case represent depths of up to 44.3 μm. The presence of these local voids suggests the possibility of air entrapment during the process of adhesive application or insufficient adhesive flow into the deeper groove structures. However, tensile lap-shear strength values indicate that this incomplete filling does not significantly impair bond performance. This phenomenon is likely attributable to the presence of sufficient adhesive penetration at groove edges and in partially filled regions, thereby facilitating effective mechanical interlocking. It is important to note that the image in question offers a two-dimensional representation of a polished specimen. The voids observed correspond to isolated pockets and do not extend along the entire groove.

**Figure 9:**
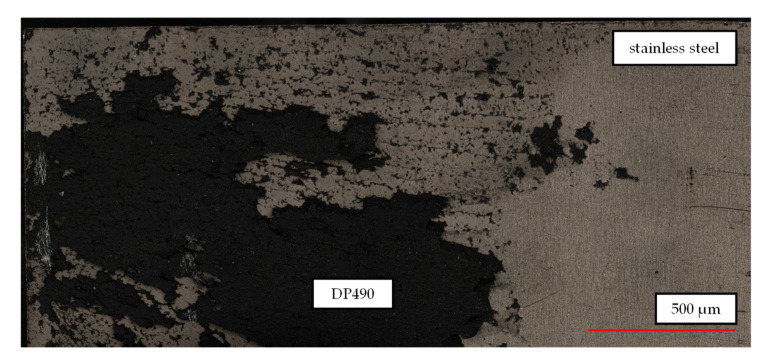
Macroscopic view of the SC stainless-steel surface with DP490 adhesive (specimen 2-2).

The failure analysis reveals that the DP490 adhesive predominantly exhibits mixed failure modes for the SC, SA, and LS surface treatments. As demonstrated in Figure 9, significant regions of DP490 exhibit adhesion to the steel substrate, while separation occurs at the interface with the CFRP in other areas. This indicates a combination of cohesive and interfacial failure, likely governed by local differences in adhesion quality or surface characteristics. In contrast, for the SG and SB surfaces, adhesive failure on the steel substrate is more frequently observed.

The SG300 adhesive predominantly exhibits cohesive failure modes, as well as, in several areas, thin-layer cohesive failure modes on the steel or CFRP side. Additionally, two specimens with SG and one with SB surface treatment exhibit clear adhesive failure. Furthermore, the MA8110 exhibits a propensity towards thin-layer cohesive failure, characterized by the significant presence of adhesive residue on the CFRP surface.

## 4. Conclusions

In this study, a specific bonding of stainless-steel connection points on a CFRP chassis was investigated at the coupon level. Firstly, the influence of three surface treatment methods (sandpaper grinding—SG, sandblasting—SB, surface cleaning—SC) on the quality of bonding with three structural adhesives (DP490, SG300, and MA8110) was investigated. The adhesive that showed high lap-shear strength combined with adequate processability was then further investigated with a sandblasted surface that had undergone an additional coating (SA) and a laser-structured surface (LS). The steel surface roughness before and after the surface treatment was characterized both qualitatively through microscopic examinations and quantitatively through roughness measurements. The findings revealed that, in contrast to the reference surface (RF), the laser structuring resulted in the most substantial enhancement in surface roughness Ra, with an increase from 1.84 ± 0.04 µm to 88.8 ± 12.2 µm. This was followed by sandblasting (3.71 ± 0.15 µm), sandpaper grinding (2.78 ± 0.14 µm), and the sandblast coating method (1.94 ± 0.34 µm). The cleaning of the surface resulted in a reduction of Ra to 1.60 ± 0.07 µm.

In order to analyze the bond strength, the tensile lap-shear strength was determined using double-lap specimens. The findings indicate that, in contrast to the outcomes observed in previous studies that attributed benefits to surface roughening treatments such as sandblasting and sandpaper grinding, the highest tensile lap-shear strength of DP490 and MA8110 was not achieved with roughened surfaces. Instead, this was achieved on surfaces that were simply cleaned, while the tensile shear strength of SG300 shows similar values across the different surface treatments. Among the three adhesives that were examined, DP490 exhibited the highest performance, with strength values reaching 29.3 ± 1.16 MPa. The lower strengths observed with roughened surfaces can be attributed to incomplete penetration of the adhesives into the surface recesses, resulting in the formation of small voids between the steel and the adhesive surface. These voids were found to be the primary cause of an earlier adhesive failure at the interface between the adhesive and the metal. Microscopic examination of the LS surfaces revealed that the adhesive was unable to fully penetrate the grooves created by the laser treatment. However, this incomplete penetration did not significantly impact the bond strength, suggesting that partial penetration was sufficient to maintain adequate bonding between the surfaces. SA produced results that were almost identical to those of SC, which can be attributed to their similar surface textures.

Failure analysis revealed that the DP490 adhesive predominantly exhibited mixed failure modes for the SC, SA, and LS surface treatments. For the SG and SB surfaces, adhesive failure on the steel substrate was more frequently observed. The SG300 adhesive predominantly exhibited cohesive failure modes, as well as, in several areas, thin-layer cohesive failure modes on the steel or CFRP side. Furthermore, two specimens with SG and one with SB surface treatment exhibited clear adhesive failure. Additionally, the MA8110 adhesive demonstrated a proclivity for thin-layer cohesive failure, as evidenced by the substantial presence of adhesive residue on the CFRP surface.

These findings suggest that in this specific application, surface cleanliness may be more critical than surface roughness for achieving optimal adhesive bonding. This has important implications for later industrial applications, as it indicates that simpler and more cost-effective surface preparation methods might be preferable to more complex, often time-consuming and expensive treatments.

However, it is important to acknowledge the limitations of this study. The utilization of a singular type of CFRP laminate and a solitary stainless-steel grade is a factor that serves to limit the generalizability of the findings to alternative material combinations. Furthermore, it is important to note that all experiments were conducted under constant temperature and humidity in a controlled laboratory setting, without accounting for environmental factors such as UV exposure, moisture, or temperature fluctuations. The present study concentrated exclusively on quasi-static tensile lap-shear testing, without addressing long-term durability aspects such as fatigue behavior, environmental aging, or creep.

Although the bonded chassis will undergo hydroforming impulse tests to evaluate service-life performance under near-realistic conditions, these tests will not provide material-specific fatigue life curves. Therefore, future work should include comprehensive coupon-level fatigue testing, vibrational loading studies, and environmental exposure assessments to fully characterize the long-term performance of adhesive bonds in agricultural machinery. Such investigations will be essential to develop robust and reliable bonding guidelines for industrial use, particularly in applications where adhesive bonds are exposed to harsh and variable operating environments.

In addition to these efforts, future research could also investigate the potential of advanced surface pretreatment methods, such as plasma activation or chemical etching, to further enhance adhesion performance at the stainless-steel interface. Furthermore, the expansion of the study to additional metal–CFRP pairings would assist in determining whether analogous adhesion trends and failure behaviors are observed across disparate material systems. Finally, further investigation into the combination of structural adhesives and selected surface treatments and materials may reveal whether the observed strength relationships are consistent across different adhesive chemistries. This would contribute to more generalized bonding recommendations.

## Figures and Tables

**Table 1:** Information about the adhesives (from the technical data sheet).

	DP490	MA8110	SG300-15
Producer	3M Scotch-Weld	Plexus	SCIGRIP
Resin type	Epoxy	Methacrylat	Methacrylat
Materials	CFRP	CFRP/steel	aluminum
Curing at RT	7 days	2.5–3 h	50 min
Optimal bonding gap (mm)	0.05–0.15	0.75–12.7	0.5
Tensile lap strength (MPa)	36	19.3	16–19
Failure mode	Fiber-tear failure	Cohesive failure	-

## Data Availability

The raw data supporting the conclusions of this article will be made available by the authors on request.
